# Endothelial NEDD4L exacerbates acute lung injury by targeting A20 for ubiquitination degradation

**DOI:** 10.1186/s12931-026-03655-w

**Published:** 2026-04-06

**Authors:** Caijuan Huan, Fang Jia, Pan Liu, Zhiyong Xu, Yueli Shi

**Affiliations:** 1 Department of Respiratory Disease, Thoracic Disease Center, The First Affiliated Hospital of Zhejiang University School of Medicine, Hangzhou, Zhejiang 310003 China; 2 Department of Breast Surgery, Zhejiang Cancer Hospital, Hangzhou Institute of Medicine (HIM), Chinese Academy of Sciences, Hangzhou, Zhejiang 310022 China; 3 Department of Pathology, Zhejiang Cancer Hospital, Hangzhou Institute of Medicine (HIM), Chinese Academy of Sciences, Hangzhou, Zhejiang 310022 China

**Keywords:** Acute lung injury, Endothelial dysfunction, NF-κB/MAPK signaling, NEDD4L, Ubiquitination, A20 (TNFAIP3)

## Abstract

Acute lung injury (ALI) and acute respiratory distress syndrome (ARDS) are life-threatening critical diseases driven by uncontrolled inflammation and endothelial dysfunction, with limited effective targeted therapies available. NEDD4L, an E3 ubiquitin ligase, has been linked to ALI pathogenesis, but its role in endothelial cells remains undefined. Here, we explored the function and mechanism of endothelial NEDD4L in ALI. Single-cell RNA sequencing of a sepsis-induced ALI mouse model and in vitro assays revealed marked upregulation of NEDD4L in endothelial cells under ALI-related pathological stimuli (LPS, TNFα, H₂O₂). Endothelial cell-specific NEDD4L knockdown (NEDD4Lᴷᴰ) in mice significantly alleviated LPS-induced lung injury, as evidenced by reduced pathological damage, inflammatory infiltration, cytokine release and improved survival, and similar protective effects were observed in a CLP-induced ALI model. In vitro experiments confirmed that NEDD4L knockdown suppressed TNFα-induced endothelial activation and leukocyte adhesion. Mechanistically, NEDD4L directly bound to the anti-inflammatory protein A20, promoting its polyubiquitination and proteasomal degradation, which impaired A20-mediated inhibition of the JNK/p38/NF-κB pathway and upregulated the expression of pro-inflammatory cytokines and adhesion molecules. Rescue assays verified that A20 overexpression partially reversed NEDD4L induced pro-inflammatory effects. Collectively, our study identifies a novel NEDD4L-A20 axis in endothelial cells that contributes to ALI progression by modulating inflammatory signaling and endothelial dysfunction. Targeting this regulatory axis may provide a potential precise and cell-selective therapeutic strategy for alleviating ALI/ARDS.

**Supplementary Information:**

The online version contains supplementary material available at 10.1186/s12931-026-03655-w.

## Introduction

Acute lung injury (ALI) is a life-threatening syndrome characterized by acute respiratory failure from diverse etiologies, presenting with refractory hypoxemia, non-cardiogenic pulmonary edema, and acute respiratory distress [1]. In the absence of timely and effective intervention, ALI can progress to Acute Respiratory Distress Syndrome (ARDS) and subsequent multiple organ dysfunction syndrome (MODS), with an overall mortality rate of approximately 45% [2]. Despite advances in supportive care (e.g., lung-protective ventilation, fluid resuscitation), no specific therapies exist [3, 4]. Elucidating key regulatory networks in ALI pathogenesis is urgently needed to improve outcomes.

The core pathology of ALI is uncontrolled inflammation, driven by aberrant leukocyte recruitment and infiltration into lung parenchyma via interactions with pulmonary endothelium [5]. Endothelial cells act as critical inflammatory regulators: pro-inflammatory cytokines such as TNFα induce endothelial cells activation, upregulating adhesion molecules (ICAM-1, VCAM-1) and chemokines (CCL2, CCL5) to promote leukocyte extravasation, while disrupting endothelial barrier function and increasing vascular permeability [6–8]. This forms a vicious cycle of endothelial dysfunction, leukocyte infiltration, and sustained inflammation, highlighting endothelial cells as therapeutic targets.

NEDD4L/NEDD4-2, a key member of the NEDD4 family that exerts E3 ubiquitin ligase activity, has been identified to be involved in the pathogenesis of various inflammatory diseases such as asthma and colitis [9–11]. NEDD4L has also attracted extensive attention in ALI research, with existing studies primarily focusing on alveolar epithelial cells (AECs). These studies demonstrate that upregulated NEDD4L induces ubiquitin-dependent degradation of the epithelial sodium channel (ENaC) and Na⁺,K⁺-ATPase, directly impairing alveolar fluid clearance and solidifying its critical pro-ALI role [12]. Notably, endogenous lipid mediators (PTRC1) and therapeutic agents (dexmedetomidine) alleviate ALI by suppressing NEDD4L, thereby preserving ENaC and Na⁺,K⁺-ATPase function [13, 14]. In contrast, hypercapnia-related JNK signaling activation upregulates NEDD4L activity, accelerating ENaC degradation to exacerbate lung injury [15]. These findings validate targeting NEDD4L for ALI therapy and underscore the need to explore its function across diverse cell types.

Beyond AECs, emerging evidence highlights NEDD4L as a central regulator of vascular endothelial homeostasis and dysfunction. For instance, G protein-coupled receptors such as PAR1mediate NEDD4L tyrosine phosphorylation at the Y485 site via c-Src kinase, activating its E3 ligase activity to trigger p38 MAPK signaling and disrupt endothelial barrier integrity [16]. In different pathological contexts, NEDD4L exerts distinct functions in the endothelial by ubiquitinating and degrading specific substrates: in a diabetic atherosclerosis model, NEDD4L inhibits endothelial injury by degrading RASGRP2 [17]; in diabetic retinopathy, a high-glucose environment upregulates NEDD4L to promote IKK-2-dependent IκBα degradation, activating NF-κB and leading to vascular inflammation, oxidative stress, and increased permeability [18]; in a coronary heart disease model, SGK1 promotes endothelial cell ferroptosis by activating the NEDD4L–NF-κB axis [19]. Despite its established role as a pivotal endothelial regulator, the specific molecular mechanisms of endothelial NEDD4L during ALI remain to be clarified.

Herein, we investigated the function of NEDD4L in endothelial cells during ALI. Results showed NEDD4L is upregulated in endothelial cells in ALI. Mechanistically, NEDD4L ubiquitinates and degrades the anti-inflammatory protein A20 (TNFAIP3), abrogating its inhibition of TNFα-induced NF-κB and MAPK pathways, thereby promoting endothelial activation and inflammatory infiltration to exacerbate ALI. This identifies a novel molecular axis of NEDD4L-A20 in ALI pathogenesis, providing experimental basis for endothelial-targeted therapies.

## Materials and methods

### Cell culture and treatments

Human umbilical vein endothelial cells (HUVECs) were cultured in endothelial cell growth medium (EGM-2, Lonza) supplemented with 10% FBS and 1% PSG. HEK293T cells were maintained in Dulbecco’s modified Eagle’s medium (DMEM, Gibco) with the addition of 10% FBS and 1% PSG. Both cell lines were cultured at 37 °C in a humidified incubator with 5% CO₂. HUVECs were treated with TNFα (0–10 ng/mL, PeproTech, 300-01A), LPS (0–1 μg/mL, Sigma-Aldrich,L2880), or H₂O₂ (0–150 μM, Sigma-Aldrich, H1009) for the indicated time periods (0–24 h). For proteasome inhibition, cells were pretreated with MG132 (10 μM, MedChemExpress, HY-13259) for 8 h.

### Animal experiments

C57BL/6 J wild-type (WT) mice (6–8 weeks old) were used in all experiments. Mice were housed under specific pathogen-free conditions with a 12-h light/dark cycle. All animal experimental studies were conducted in accordance with the guidelines of the Institutional Animal Care and Use guidelines and were approved by the Animal Care and Use Committee of the Zhejiang University School of Medicine. Endothelial-specific NEDD4L-knockdown AAV6 vectors (Tie2 promoter-driven shRNA) and empty AAV6 controls were packaged, stored at −80℃ in dark, and thawed gently on ice before use. Mice were anesthetized with isoflurane, and 50 μL viral solution (1 × 10^11^ vg/mouse for NEDD4L-shRNA AAV6; equal empty AAV6 for control) was tracheally instilled. Three weeks later, 3–5 mice/group were sacrificed. Pulmonary endothelial cells were isolated by CD31⁺ magnetic sorting. NEDD4L expression was detected by WB (β-actin as internal reference), with ≥ 50% knockdown efficiency required for LPS modeling. Mice were anesthetized and challenged with LPS (5 mg/kg) via intratracheal instillation. Control mice received sterile saline. Mice were sacrificed at 24 h post-LPS challenge. Bronchoalveolar lavage fluid (BALF) was collected by flushing the lungs with 1 mL of sterile PBS, while lung tissues were harvested for subsequent histological analysis, wet/dry weight ratio measurement, and protein/mRNA extraction. Total leukocyte counts in BALF were determined using a hemocytometer, and total protein concentration was measured with a BCA protein assay kit (Thermo Fisher Scientific). Cytokine levels, including IL-6, IL-1β, and TNFα, were quantified by ELISA (R&D Systems) following the manufacturer’s instructions. For assessment of pulmonary edema, lung tissues were weighed immediately after harvest to record the wet weight, then dried at 60 °C for 48 h to obtain the dry weight; the wet/dry weight ratio was subsequently calculated. For histological evaluation, lung tissues were fixed in 4% paraformaldehyde, embedded in paraffin, sectioned into 5 μm slices, and stained with hematoxylin and eosin (H&E). Images of the stained sections were acquired using a light microscope (Olympus).

### Analysis of transcriptome data

Transcriptome data sets of human samples GSE2638 and GSE279126 were downloaded from the GEO database. GSE2638 was microarray derived gene expression profiling, while GSE279126 was RNA-seq derived gene expression profiling, which was further analyzed in the form of FPKM (Fragments Per Kilobase of transcript per Million mapped reads). After log-normalization of the expression data, differential expression analysis was performed using the limma R package (version 3.64.1). A design matrix was constructed based on experimental groups, and linear models were fitted for each gene using the lmFit function. Empirical Bayes moderation of the standard errors was applied using the eBayes function to improve statistical power.

### Analysis of scRNA-seq data

The scRNA-seq data set of mouse lung tissue GSE207651 was downloaded from the GEO database. Analysis was performed using the Seurat R package (version 5.3.0). The low-quality cells were removed if they expressed < 300 genes/cell, UMI counts < 500 or mitochondrial UMI counts greater than 20%. The CellCycleScoring and SCTransform functions were performed with default parameters before integration, in order to normalize the data and to regress out irrelevant sources of variation including those derived from mitochondrial expression. The harmony R package (version 1.2.3) was used to remove batch effects between samples. The cells after quality control were grouped with the FindNeighbors and FindClusters functions with a resolution of 0.5. The clusters were then visualized after RunUMAP function. Cell types of each cluster were annotated by the expression of canonical markers. To perform differential expression analysis, the gene expression of cells from different groups was compared using the Wilcox test with the Bonferroni false discovery rate (FDR) correction.

### Bioinformatic analysis of protein–protein interactions

Protein–protein interaction (PPI) network: The PPI network of A20 (TNFAIP3) was constructed using the BioGRID database (https://thebiogrid.org/). E3 ligase interaction network: The E3 ligase interaction network centered on A20 was generated using the UbiBrowser database (http://ubibrowser.bio-it.cn/). The top 10 E3 ligases with the highest confidence scores for interaction with A20 were identified.

### Lentivirus packaging and infection

Lentiviral vectors included pLVX-Puro-NEDD4L, pLVX-Neo-A20, pLKO-shRNA-NEDD4L and their controls (pLVX-Puro, pLVX-Neo, pLKO-shRNA-NC); the sequences of the shRNA targeting NEDD4L are provided in Supplementary Table 1. 293 T cells were seeded at 2 × 10⁶ cells/mL in 10 cm dishes and cultured to 70%–80% confluency. Transfection complexes (8 μg vector, 4 μg psPAX2, 4 μg pMD2.G, 24ul P3000, 30 μL Lipofectamine 3000 in 1 mL Opti-medium) were incubated for 15 min and added to cells. Medium was replaced 6–8 h later. Viral supernatants collected at 48 post-transfection were filtered (0.22 μm), aliquoted and stored at −80℃. NEDD4L and A20 lentiviruses for co-overexpression were packaged separately.

HUVECs were seeded at 1 × 10^5^ cells/mL in 6-well plates to 50%–60% confluency, then infected with lentiviruses (*n* = 3 replicates/group): NEDD4L overexpression, NEDD4L knockdown, NEDD4L/A20 co-overexpression (mixed viruses) and their controls. Polybrene (8 μg/mL) was added to enhance infection. After 24 h, virus-containing medium was replaced with fresh complete medium, and cells were cultured for another 48–72 h for gene expression analysis.

### Quantitative Real-Time PCR (qRT-PCR)

Total RNA was extracted from cells using the RNA-Quick Purification Kit (Yishan). The concentration and purity were measured with a NanoDrop spectrophotometer (Thermo Fisher). cDNA was synthesized from 1 μg total RNA using the PrimeScript RT reagent kit (TaKaRa) according to the manufacturer’s instructions. qRT-PCR was performed with SYBR Green PCR Master Mix (Vazyme), and the sequences of the qPCR primers are provided in Supplementary Table 2. Relative mRNA expression levels were calculated using the 2⁻ΔΔCt method, normalized to 18S rRNA.

### Western blot analysis

Cells were lysed in RIPA buffer (Beyotime) containing protease and phosphatase inhibitors (Roche). Protein concentration was determined using a BCA protein assay kit (Beyotime). Equal amounts of protein (30–50 μg) were separated by SDS-PAGE and transferred to PVDF membranes (Millipore). Membranes were blocked with 5% non-fat milk in TBST for 1 h, then incubated with indicate primary antibodies overnight at 4 °C. After washing, membranes were incubated with HRP-conjugated secondary antibodies (Cell Signaling Technology) for 1 h at room temperature. Protein bands were visualized using an ECL chemiluminescence detection kit (Millipore) and quantified using ImageJ software.

### Co-Immunoprecipitation (Co-IP) assay

HUVECs were lysed in IP buffer (Beyotime) supplemented with protease and phosphatase inhibitors. Anti-NEDD4L antibody or IgG isotype control was pre-incubated with Protein A/G magnetic beads at 4 °C for 10 min to form antibody-bead complexes. The prepared complexes were then incubated with the above cell lysates at 4 °C overnight with gentle rotation. After extensive washing, the bead-bound proteins were eluted and subjected to Western blot analysis.

HEK293T cells were transiently transfected with Flag-A20 and HA-NEDD4L plasmids. At 48 h post-transfection, cells were lysed with IP buffer containing protease and phosphatase inhibitors. Anti-Flag antibody or IgG isotype control was pre-incubated with Protein A/G magnetic beads at 4 °C for 10 min. The cell lysates were then added to the antibody-bead complexes and incubated at 4 °C overnight with gentle rotation. Following several washes, the bead-bound proteins were collected and analyzed by Western blot.

### Ubiquitination assay

HEK293T cells were transfected with Flag-A20, Myc-Ub, and HA-NEDD4L or knockdown by shNEDD4L lentivirus. Cells were treated with MG132 (10 μM) for 8 h prior to harvest. Lysates were immunoprecipitated with anti-Flag antibody, and the immunoprecipitates were analyzed by Western blot using anti-Myc antibody to detect ubiquitinated A20.

### Flow cytometry

HUVECs were harvested, washed with PBS, and stained with anti-ICAM1-PE (BD Biosciences) or anti-VCAM1-FITC (BD Biosciences) antibodies for 30 min at 4 °C. Cells were washed, and fluorescence intensity was measured using a FACSCanto II flow cytometer (BD Biosciences). Data were analyzed using FlowJo software.

### HL-60 cell adhesion assay

HUVECs were seeded in 24-well plates and infected with the indicated lentivirus. HL-60 cells were labeled with Calcein-AM (Invitrogen) for 30 min at 37 °C. Labeled HL-60 cells were added to the HUVEC monolayer and incubated for 1 h at 37 °C. Non-adherent cells were removed by washing with PBS. Adherent cells were visualized using a fluorescence microscope (Olympus), and the number of adherent cells per field was counted.

### Statistical analysis

All data were expressed as the mean ± standard deviation (SD) derived from at least three independent experimental replicates; statistical significance of intergroup differences was assessed using the Student’s t-test for comparisons between two independent groups, one-way analysis of variance (ANOVA) for multiple-group comparisons, and the log-rank test for survival curve analysis, with a value of *p* < 0.05 being regarded as statistically significant. *p < 0.05, **p < 0.01, ***p < *0.001.*^*ns*^*p* ≥ *0.05.*

## Results

### Upregulation of NEDD4L in endothelial cells during ALI and pro-inflammatory challenge

**Fig. 1:**
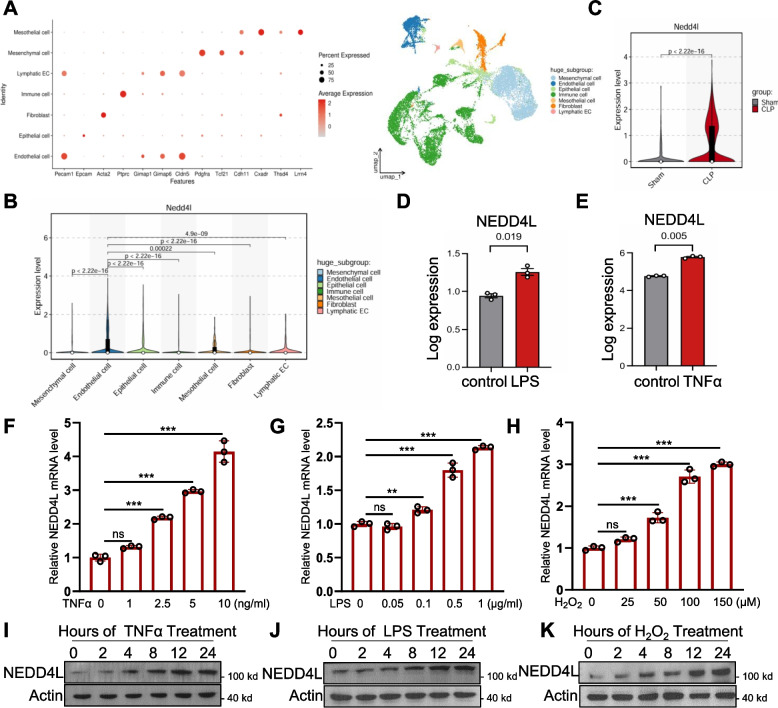
Acute lung injury and related stimuli induce NEDD4L expression in endothelial. **A** Dot plot of canonical marker gene expression (left) and UMAP visualization (right) based on single-cell RNA sequencing (scRNA-seq) data from dataset GSE207651 showing the annotated cell clusters.** B** Violin plots showing the distribution of NEDD4L expression across distinct cell clusters identified in the scRNA-seq dataset.** C** Violin plot of NEDD4L expression in lung endothelial cells from Sham and CLP groups based on scRNA-seq data.** D** Analysis of NEDD4L expression profiles in GSE279126 dataset of endothelial cells treated with or without LPS.** E** Analysis of NEDD4L expression profiles in GSE2638 dataset of endothelial cells treated with or without TNFα. **F–H** Dose-dependent effects of TNFα (**F**), LPS (**G**), and H₂O₂ (**H**) on NEDD4L mRNA expression measured by qRT-PCR (*n* = 3). HUVECs were treated with increasing concentrations of stimuli for 12 h. Statistical analysis was performed with one-way ANOVA, and data are expressed as mean ± SD. **I-K** Western blot analysis of NEDD4L protein expression in HUVECs treated with TNFα (10 ng/mL; **I**), LPS (1 μg/mL; **J**), or H₂O₂ (150 μM; **K**) over a 24 h time course. Actin serves as a loading control. Representative blots from three independent experiments are shown

To investigate the potential role of NEDD4L in ALI, we first analyzed single-cell RNA sequencing (scRNA-seq) data from the GSE207651 dataset, which was derived from a mouse model of sepsis-induced ALI (cecal ligation and puncture, CLP). Dot plot and UMAP visualization revealed distinct cell clusters in the lung tissue, including mesenchymal cells, endothelial cells, epithelial cells, immune cells, mesothelial cells, fibroblasts, and lymphatic endothelial cells (Fig. 1A). Violin plot analysis demonstrated that NEDD4L is expressed across all these cell populations, with particularly notable expression in endothelial and epithelial cells (Fig. 1B). Further, scRNA-seq analysis of lung endothelial cells from Sham and CLP groups showed that NEDD4L expression was significantly higher in the CLP group compared with the Sham group (Fig. 1C).

To validate these observations, we analyzed public transcriptomic datasets and found that NEDD4L expression was significantly upregulated in endothelial cells treated with LPS and TNFα (Fig. 1D-E). To further recapitulate the in vitro pathological microenvironment of ALI, HUVECs were treated with exogenous stimuli (LPS for inflammation, H₂O₂ for oxidative stress, TNFα as pro-inflammatory cytokine) to mimic ALI-associated inflammation and oxidative stress. Results showed dose-dependent upregulation of NEDD4L mRNA expression upon respective stimulations (Fig. 1F–H). Western blot analysis further demonstrated that NEDD4L protein levels were also upregulated in response to these pro-inflammatory and oxidative stress stimuli over a time course (Fig. 1I–K).

### Endothelial NEDD4L knockdown attenuates LPS-induced ALI

**Fig. 2:**
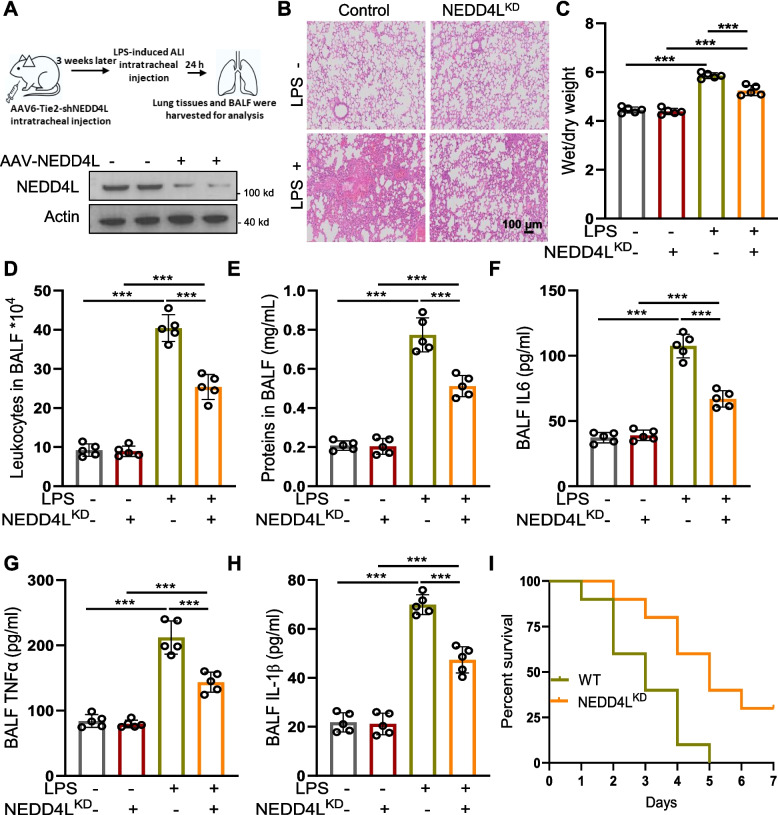
NEDD4L knockdown attenuates LPS-induced acute lung injury in mice. **A** Schematic of AAV-mediated NEDD4L knockdown strategy and representative Western blot verifying NEDD4L knockdown efficiency in isolated mice lung endothelial cells, with β-actin as a loading control. **B** H&E staining of lung sections from WT and NEDD4L^KD^ mice with or without LPS treatment (scale bar = 100 μm). **C** Lung wet/dry weight ratio in each group, reflecting pulmonary edema severity. **D-H** Quantification of inflammatory parameters in Bronchoalveolar Lavage Fluid (BALF): total leukocyte count (**D**), total protein concentration (**E**), IL-6 (**F**), TNFα (**G**), and IL-1β (H) levels. **C**–**H**, data are presented as mean ± SD (*n* = 5/group), and statistical analysis was performed using one-way ANOVA. **I** Kaplan–Meier survival curve of WT and NEDD4L.^KD^ mice following LPS challenge (n = 10 per group)

To specifically assess the pathological significance of endothelial NEDD4L upregulation in ALI, we constructed an EC-specific NEDD4L knockdown (NEDD4L^KD^) mouse model via intratracheal instillation of AAV6 carrying the Tie2 promoter. Sorting and Western blot validation confirmed that NEDD4L expression was significantly suppressed in mouse lung ECs (Fig. 2A). We then induced ALI via intratracheal instillation of LPS and evaluated lung pathological changes and inflammatory responses. H&E staining showed that LPS caused severe lung injury in WT mice, which was characterized by obvious alveolar wall thickening, structural destruction, hemorrhage, and massive inflammatory cell infiltration; these pathological damages were significantly alleviated in NEDD4L^KD^ mice (Fig. 2B). NEDD4L knockdown also reduced the lung wet/dry ratio (Fig. 2C), BALF leukocyte count, total protein concentration, and pro-inflammatory cytokine levels (IL-6, TNFα, IL-1β) (Fig. 2D–H). To elucidate the therapeutic potential of NEDD4L knockdown in ALI, we established a survival analysis model by intratracheally instilling LPS at 50 mg/kg, and subsequent analysis indicated that NEDD4L ablation conferred a survival advantage (Fig. 2I). Additionally, NEDD4L knockdown mitigated LPS-induced oxidative stress, restoring SOD/CAT activity and GSH levels while reducing MDA accumulation (Supplementary Fig. 1A-D).

To further confirm the protective effect of endothelial NEDD4L knockdown across different ALI etiologies, we established a CLP-induced ALI model. Consistent with the findings from the LPS-induced ALI model, H&E staining showed that CLP-induced lung pathological damage was significantly alleviated in NEDD4L^KD^ mice compared to WT mice (Supplementary Fig. 2 A). Similarly, NEDD4L knockdown reduced the lung wet/dry weight ratio (Supplementary Fig. 2B) and decreased BALF protein concentration, leukocyte count, and pro-inflammatory cytokine (IL-6, TNFα, IL-1β) levels (Supplementary Fig. 2C-G). Collectively, these findings from both LPS and CLP-induced ALI models confirm that endothelial NEDD4L knockdown alleviates ALI, highlighting its potential for ALI therapy.

### NEDD4L deficiency attenuates endothelial activation and inflammatory cell infiltration

**Fig. 3:**
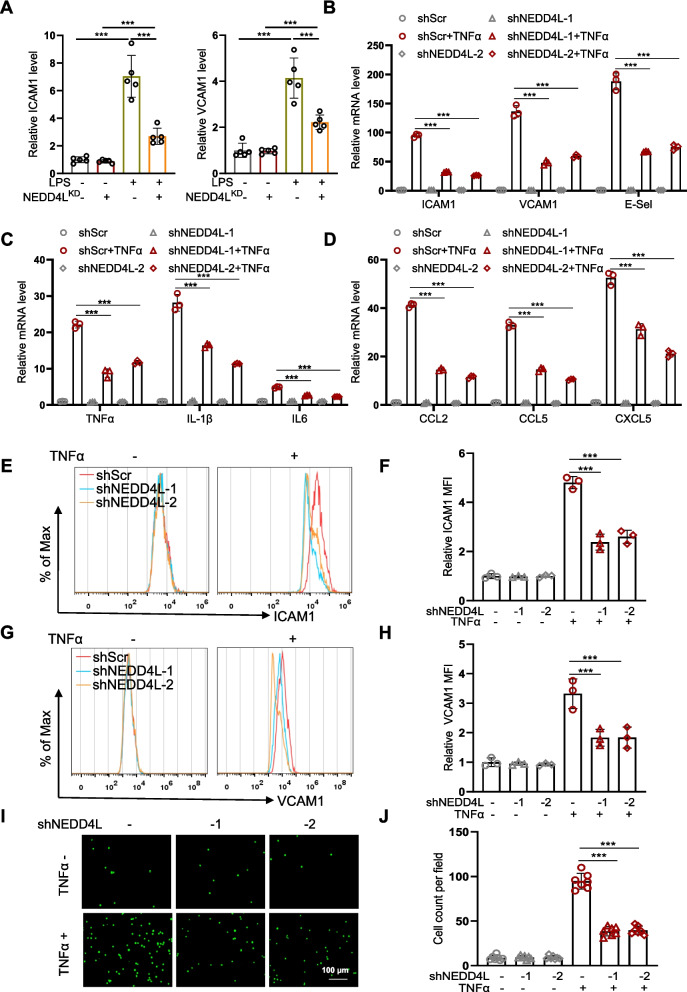
NEDD4L knockdown inhibits TNFα-induced inflammatory and adhesion molecules expression in endothelial cells. **A** Relative mRNA levels of the adhesion molecules ICAM1 and VCAM1 in lung tissue from WT and NEDD4LKD mice, with or without LPS stimulation (*n* = 5). Statistical analysis was performed with one-way ANOVA, and data are expressed as mean ± SD. **B-D** Relative mRNA levels of adhesion molecules (ICAM1, VCAM1, E-Selectin) (**B**), pro-inflammatory cytokines (TNFα, IL-1β, IL-6) (**C**) and chemokines (CCL2, CCL5, CXCL5) (D) in HUVECs transfected with shScr or shNEDD4L, with or without TNFα treatment (*n* = 3). Statistical analysis was performed with two-way ANOVA, and data are expressed as mean ± SD. **E–H** Flow cytometry analysis of cell surface ICAM1 (**E**, **F**) and VCAM1 (**G**, **H**) protein expression in HUVECs transfected with shScr or shNEDD4L, with or without TNFα treatment. Histograms show representative staining, and bar graphs summarize relative mean fluorescence intensity (MFI, *n* = 3). Statistical analysis was performed with one-way ANOVA, and data are expressed as mean ± SD. **I, J** HL-60 cell adhesion assay: **I** Representative fluorescent images of HL-60 cells (green) adhering to HUVECs under different conditions (scale bar = 100 μm). **J** Quantification of adherent HL-60 cells per field (*n* = 7). Statistical analysis was performed with one-way ANOVA, and data are expressed as mean ± SD

Leukocyte extravasation into inflamed lung tissue is initiated by leukocyte-endothelial adhesion. We first quantified ICAM-1 and VCAM-1 transcript levels in lung tissue from WT and NEDD4L^KD^ mice after LPS challenge. NEDD4L deficiency significantly attenuated LPS-induced upregulation of both adhesion molecules (Fig. 3A). To validate this in vitro, we stimulated HUVECs with TNFα and modulated NEDD4L expression. NEDD4L knockdown suppressed TNFα-induced mRNA expression of adhesion molecules (ICAM1, VCAM1, E-selectin), pro-inflammatory cytokines (TNFα, IL-1β, IL-6), and chemokines (CCL2, CCL5, CXCL5) (Fig. 3B–D). Flow cytometry confirmed reduced cell surface ICAM1 and VCAM1 protein levels (Fig. 3E–H). Conversely, NEDD4L overexpression potentiated TNFα-mediated upregulation of these molecules (Supplementary Fig. 3A-C). Functional HL-60 adhesion assays showed that NEDD4L knockdown inhibited TNFα-induced leukocyte adhesion to HUVECs (Fig. 3I–J), while overexpression enhanced it (Supplementary Fig. 3D-E). These data demonstrate that NEDD4L promotes endothelial activation and leukocyte recruitment by regulating the expression of adhesion molecules, pro-inflammatory cytokines, and chemokines, thereby defining its critical pathological role in acute lung injury.

### NEDD4L enhances TNFα-mediated signaling pathway activation by inhibiting A20

**Fig. 4:**
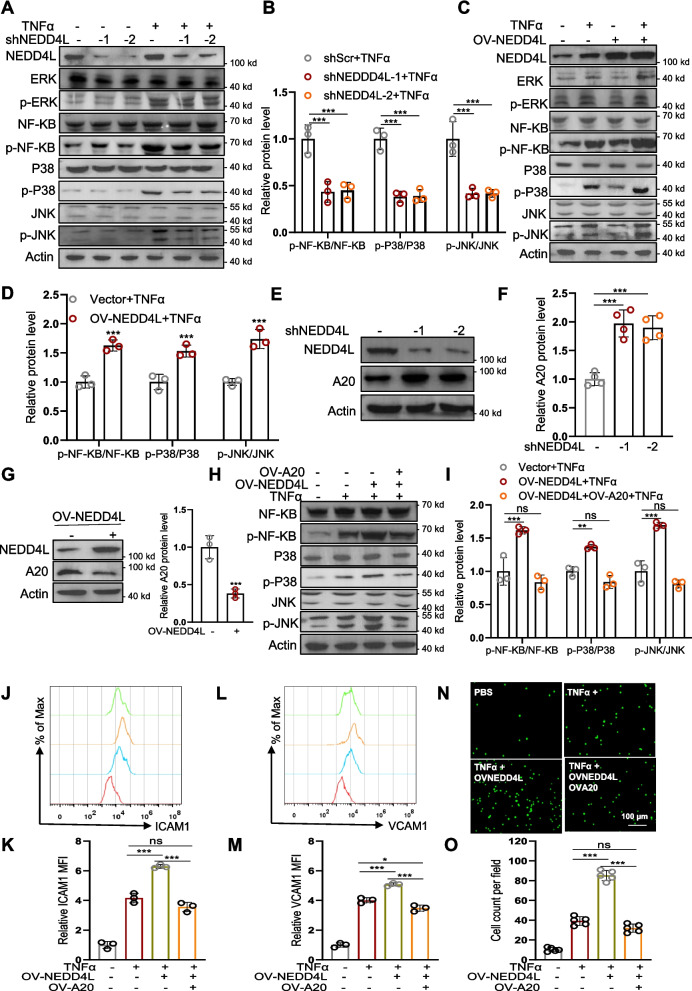
NEDD4L promotes TNFα-induced inflammatory responses in endothelial cells via regulation of the A20-mediated MAPK/NF-κB signaling pathway. **A**, **B** Western blot analysis (**A**) and quantification (**B**, *n* = 3) of phosphorylated/total protein levels of NF-κB, p38 MAPK, JNK, and ERK in HUVECs transfected with shScr or shNEDD4L, with or without TNFα stimulation. Statistical analysis was performed with two-way ANOVA, and data are expressed as mean ± SD. **C**, **D** Western blot analysis (**C**) and quantification (**D**, *n* = 3) of phosphorylated/total protein levels of NF-κB, p38 MAPK, JNK, and ERK in HUVECs transfected with vector or OV-NEDD4L, with or without TNFα stimulation. Statistical analysis was performed with two-way ANOVA, and data are expressed as mean ± SD. **E**, **F** Western blot analysis (**E**) and quantification (**F**, *n* = 4) of A20 protein levels in HUVECs transfected with shScr or shNEDD4L. Statistical analysis was performed with one-way ANOVA, and data are expressed as mean ± SD.** G** Western blot analysis and quantification of A20 protein levels in HUVECs transfected with empty vector or OV-NEDD4L (*n* = 3). Statistical analysis was performed using Student's t-test, and data are expressed as mean ± SD. **H**, **I** Western blot analysis (**H**) and quantification (**I**, *n* = 3) of phosphorylated/total protein levels of NF-κB, p38 MAPK, and JNK in HUVECs transfected with OV-NEDD4L, OV-A20, or both, with or without TNFα stimulation. Statistical analysis was performed with two-way ANOVA, and data are expressed as mean ± SD. **J**, **K** Flow cytometry histograms (**J**) and quantification (**K**, *n* = 3) of relative ICAM1 MFI in HUVECs under the conditions described in (H). Statistical analysis was performed with one-way ANOVA, and data are expressed as mean ± SD. **L**, **M** Flow cytometry histograms (**L**) and quantification (**M**, *n* = 3) of relative VCAM1 MFI in HUVECs under the conditions described in (**H**). Statistical analysis was performed with one-way ANOVA, and data are expressed as mean ± SD. **N**, **O** Representative fluorescent images (**N**, scale bar = 100 μm) of HL-60 cells (green) adhering to HUVECs under the conditions described in (**H**), and quantification of adherent HL-60 cells per field (**O**, *n* = 5). Statistical analysis was performed with one-way ANOVA, and data are expressed as mean ± SD

To investigate how NEDD4L modulates TNFα-induced endothelial activation, we analyzed the phosphorylation of key downstream molecules: NF-κB (p65) and MAPK family members. TNFα stimulation increased phosphorylation of JNK, p38, ERK, and p65 in HUVECs. NEDD4L knockdown significantly reduced TNFα-induced phosphorylation of JNK, p38, and p65, while ERK phosphorylation remained unchanged (Fig. 4A, B). Conversely, NEDD4L overexpression enhanced JNK, p38, and p65 phosphorylation, with no effect on ERK (Fig. 4C, D). A20 is a well-known negative regulator of these pathways [20–22]. Based on the analysis of the GSE207651 single-cell dataset, we first confirmed that A20 is expressed in lung endothelial cells of ALI mice (Supplementary Fig. 4 A). Notably, A20 expression was significantly downregulated upon stimulation with LPS, TNFα, and H₂O₂, and its regulatory pattern was opposite to that of NEDD4L (Supplementary Fig. 4B). We therefore hypothesized that NEDD4L promotes ALI by downregulating A20. Experimental validation revealed that NEDD4L knockdown increased A20 protein levels (Fig. 4E, F), while overexpression suppressed A20 expression (Fig. 4G). Rescue experiments confirmed that A20 co-expression reversed NEDD4L’s effects on p65, p38, and JNK phosphorylation (Fig. 4H, I). Correspondingly, A20 overexpression reversed NEDD4L-driven increases in cell surface ICAM1 (Fig. 4J, K) and VCAM1 (Fig. 4L, M), and reduced leukocyte adhesion to HUVECs (Fig. 4N, O). At the mRNA level, A20 also reversed NEDD4L-mediated upregulation of pro-inflammatory cytokines, chemokines, and adhesion molecules (Supplementary Fig. 5A-C). Collectively, these results demonstrate that NEDD4L augments TNFα-induced endothelial activation by negatively regulating A20 expression, thereby enhancing the activation of the JNK/p38/NF-κB pathway, upregulating inflammatory and adhesion molecule expression, and promoting leukocyte-endothelial adhesion.

### NEDD4L mediates proteasomal degradation of A20

**Fig. 5:**
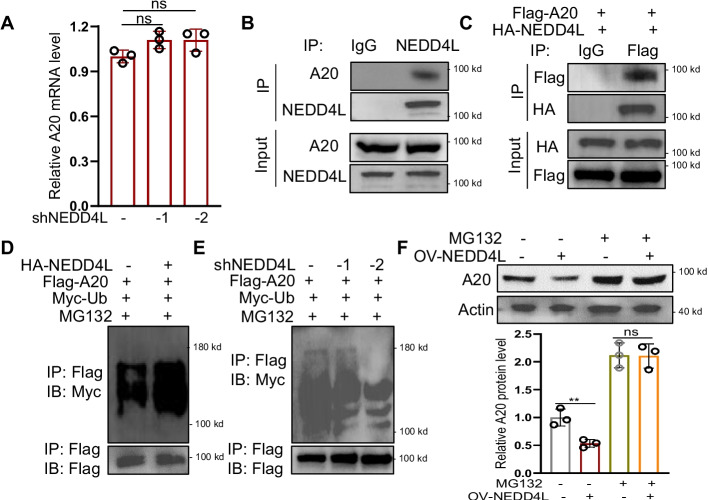
NEDD4L interacts with A20 and promotes its ubiquitination and degradation. **A** Relative A20 mRNA levels in HUVECs transfected with shScr or shNEDD4L (*n* = 3). Statistical analysis was performed with one-way ANOVA, and data are expressed as mean ± SD. **B** Endogenous CO-IP assay showing interaction between NEDD4L and A20 in endothelial cells. Representative blots from three independent experiments are shown. **C** Exogenous CO-IP assay confirming interaction between HA-tagged NEDD4L and Flag-tagged A20 in transfected 293 T cells. Representative blots from three independent experiments are shown. **D** Ubiquitination assay showing NEDD4L enhances A20 ubiquitination. HEK293T cells were transfected with Flag-A20, Myc-Ub, and HA-NEDD4L, treated with MG132, and subjected to IP with anti-Flag antibody, followed by IB with anti-Myc antibody. Representative blots from three independent experiments are shown. **E** Ubiquitination assay showing NEDD4L knockdown reduces A20 ubiquitination. HEK293T cells were transfected with Flag-A20 and Myc-Ub, treated with MG132, and subjected to IP with anti-Flag antibody, followed by IB with anti-Myc antibody. Representative blots from three independent experiments are shown. **F** Western blot and quantification showing NEDD4L promotes A20 protein degradation via the proteasome pathway. A20 protein levels were assessed in endothelial cells transfected with OV-NEDD4L, with or without MG132 treatment (*n* = 3). Statistical analysis was performed with one-way ANOVA, and data are expressed as mean ± SD

To clarify the molecular mechanism by which NEDD4L regulates A20 protein levels, we first examined its effect on A20 transcription, and as shown in Fig. 5A, NEDD4L knockdown did not alter A20 mRNA levels, indicating that NEDD4L regulates A20 at the post-translational level. Bioinformatic analysis (Supplementary Fig. 6A-C) predicted a direct interaction between NEDD4L, an E3 ubiquitin ligase, and A20; this prediction was validated by both endogenous and exogenous CO-IP assays, which confirmed the binding of NEDD4L and A20 in endothelial cells (Fig. 5B) and the interaction between HA-tagged NEDD4L and Flag-tagged A20 in transfected 293 T cells (Fig. 5C). We hypothesized that NEDD4L promotes A20 degradation via the ubiquitin–proteasome pathway, and subsequent ubiquitination assays showed that NEDD4L overexpression enhanced A20 polyubiquitination (Fig. 5D) while NEDD4L knockdown reduced it (Fig. 5E). Treatment with the proteasome inhibitor MG132 rescued NEDD4L-induced A20 degradation, confirming that NEDD4L targets A20 for proteasomal degradation (Fig. 5F). Collectively, these findings demonstrate that NEDD4L negatively regulates A20 by directly binding to it, promoting its polyubiquitination, and facilitating its degradation via the ubiquitin–proteasome pathway.

## Discussion

Endothelial-specific NEDD4L knockdown potently attenuates ALI in mice, a finding consistent with the pronounced upregulation of NEDD4L in pulmonary endothelial cells under ALI-related pathological conditions (sepsis, LPS, TNFα, oxidative stress). Integrated in vivo and in vitro data confirm endothelial NEDD4L as a critical pathological mediator of excessive inflammation and leukocyte infiltration, the core pathological features of ALI. The underlying mechanism is mediated by NEDD4L-mediated ubiquitination and proteasomal degradation of A20, which is a key negative regulator of the JNK/p38/NF-κB signaling pathway, and this process consequently enhances endothelial activation via elevated production of adhesion molecules and pro-inflammatory factors.

During inflammation progression, endothelial cells undergo a phenotypic shift from a quiescent, anti-adhesive, and low-permeability barrier to a pro-inflammatory, pro-adhesive, and hyperpermeable state [23, 24]. We observed that NEDD4L expression is significantly upregulated in response to multiple ALI-relevant stimuli, including TNFα, LPS, and H₂O₂, suggesting it may function as a conserved endothelial sensor of pathological stress. Functional assays revealed that NEDD4L overexpression not only amplifies TNFα-induced transcription of adhesion molecules (ICAM-1, VCAM-1, and E-selectin) but also enhances leukocyte adhesion. Furthermore, activated endothelial cells acquire an immune-like phenotype, releasing pro-inflammatory cytokines such as CCL2 and IL-6 that amplify leukocyte chemotaxis and perpetuate inflammatory cascades. Our data suggest that NEDD4L serve as a molecular driver of this pro-inflammatory endothelial response. Beyond these effects, growing evidence underscores endothelial cell pyroptosis as a pivotal driver in the progression of ALI/ARDS [25–28]. Given recent finding that NEDD4L upregulation promotes macrophage pyroptosis [29], we hypothesize that NEDD4L may similarly facilitate ALI progression by triggering endothelial pyroptosis, and this regulatory mechanism merits further exploration.

The NF-κB pathway is widely acknowledged as a core regulatory component in ALI pathogenesis, with its hyperactivation triggering endothelial activation, adhesion molecule upregulation, and pro-inflammatory cytokine production [6, 30, 31]. In a TNFα-challenged human endothelial cell model, NEDD4L deficiency exerted robust inhibition on TNFα-induced NF-κB activation. Moreover, JNK, ERK, and p38 of the MAPK family serve as critical mediators of ALI-related inflammation [32–34] Notably, although TNFα activates all three MAPK pathways, NEDD4L knockdown selectively blocks JNK and p38 phosphorylation without affecting ERK activation, a finding suggestive of pathway specific regulation of MAPK signaling by NEDD4L.

Building on recent evidence that JNK/p38 serve as upstream regulators of NF-κB [6], our study describes a potential dual mechanism for NEDD4L-mediated inflammatory regulation: it may directly promotes NF-κB activation while concurrently inducing the formation of the "NEDD4L–JNK/p38–NF-κB" cascade via JNK/p38 modulation, and it may potentially amplifies inflammation through the NF-κB-independent JNK/p38 pathway. This multi-hierarchical regulatory framework demonstrates that NEDD4L deficiency can comprehensively suppress endothelial inflammation, positioning NEDD4L as a critical junction in inflammatory signal crosstalk. Targeting NEDD4L may thus enable more extensive and sustained anti-inflammatory effects by blocking the cross-activation of multiple inflammatory pathways, compared to single-pathway inhibition.

As an E3 ubiquitin ligase, NEDD4L exerts its biological function through the specific recognition and ubiquitination of downstream substrates. Functional screening confirmed a direct physical interaction between NEDD4L and A20, an inflammation-suppressive protein and well characterized dual inhibitor of the NF-κB and MAPK pathways [35, 36]. Genetic polymorphisms and mutations in this protein are closely associated with the pathogenesis of diverse inflammatory disorders [37, 38]. Mechanistic assays revealed that NEDD4L induces A20 ubiquitination and proteasomal degradation, thereby releasing the inhibitory constraints of the NF-κB and MAPK pathways to drive endothelial activation. Notably, A20 was first identified in human umbilical vein endothelial cells. Consistent with our observations, previous research has validated robust inhibitory effects on endothelial activation and cytokine production [39, 40], as well as a protective role against ALI in macrophages [41, 42], underscoring extensive therapeutic potential for this protein.

Intriguingly, NEDD4L has been reported to inhibit NF-κB and p38 signaling in other cell types by targeting substrates such as MEKK2, which contrasts with its pro-inflammatory role in endothelial cells [43]. This discrepancy likely reflects the cell type specific substrate selectivity of NEDD4L, which engages distinct targets in different cellular contexts to exert opposing biological effects. While our data firmly establish A20 as a key substrate for NEDD4L in endothelial cells, the inherent substrate promiscuity of E3 ligases suggests that NEDD4L may regulate endothelial function through additional targets, warranting further investigation.

While the protective effects of NEDD4L deficiency in LPS-induced ALI are primarily ascribed to suppressed endothelial inflammatory activation, NEDD4L knockdown also attenuates LPS-induced oxidative stress, as indicated by decreased MDA levels and restored SOD, CAT and GSH activities. In ALI, inflammation and oxidative stress typically form a vicious cycle, wherein pro-inflammatory cytokines trigger ROS production that subsequently amplifies NF-κB activation [44, 45]. NEDD4L deficiency may therefore indirectly alleviate oxidative damage by inhibiting primary inflammation. Moreover, NEDD4L has been shown to downregulate NRF2 expression, indicating a potential role in suppressing antioxidant response [46]. Nevertheless, the exact mechanisms governing NEDD4L-related oxidative stress regulation remain incompletely understood. This is especially true for the respective roles of inflammation dependent and independent pathways as well as the participation of other downstream substrates, which will be prioritized in our subsequent research.

Our study has several limitations. First, we primarily used mouse models and HUVECs to investigate the role of endothelial NEDD4L in ALI. The pathological microenvironment in human ALI is more complex, and we did not validate the NEDD4L–A20 axis in clinical patient samples, limiting the translational relevance of our work. Second, although we confirmed that NEDD4L promotes A20 ubiquitination and proteasomal degradation, we did not identify the key ubiquitination sites of A20. Third, our endothelial-specific NEDD4L knockdown model was based on preventive AAV6 delivery prior to LPS/CLP challenge, which does not reflect the clinical scenario of treating established ALI. Moreover, we did not examine the long-term lung repair effects of NEDD4L inhibition.

In summary, our study identifies a novel pathological mechanism in ALI, wherein endothelial NEDD4L exacerbates inflammatory signaling via targeted A20 degradation. These findings enhance the mechanistic understanding of ALI pathogenesis and validate endothelial NEDD4L could be a promising therapeutic target. Developing small molecule inhibitors that disrupt the NEDD4L/A20 interaction may suppress excessive inflammation while preserving A20 mediated homeostatic protection in other cell types, thus offering a precise, cell-selective therapeutic strategy for ALI/ARDS.

## Conclusion

NEDD4L is markedly upregulated in endothelial cells under ALI pathological conditions, and such upregulation contributes to lung injury by triggering the ubiquitination and subsequent degradation of A20. Through attenuating the suppressive effect of A20 on the JNK/p38/NF-κB signaling pathway, NEDD4L promotes endothelial activation, enhances leukocyte recruitment, and increases pulmonary inflammatory infiltration. Our work characterizes a previously unrecognized functional interaction between NEDD4L and A20 in ALI pathogenesis, identifying endothelial NEDD4L as a potential target for future intervention against ALI and ARDS.

## Supplementary Information

Supplementary Material 1.

Supplementary Material 2.

## Data Availability

The data supporting the findings of this study are available from the corresponding author upon reasonable request.
