# Supplementary material for: Endothelial NEDD4L exacerbates acute lung injury by targeting A20 for ubiquitination degradation
Source: Respir Res. 2026 Apr 6;27:222. doi: 10.1186/s12931-026-03655-w (PMC13248476; doi:10.1186/s12931-026-03655-w)
Supplement: Supplementary file 1 — Supplementary Material 1. [file 12931_2026_3655_MOESM1_ESM.docx]

**Supplementary**

Table1. shRNA target sequences

| h-shNEDD4L-1 | CGCCTTGACTTACCTCCATAT |
| --- | --- |
| h-shNEDD4L-2 | GCGGATGAGAATAGAGAACTT |
| m-shNEDD4L-1 | CCAGAGAGTTTAAGCAGAAAT |

Table 2. qPCR primers

| hICAM1-F | ATGCCCAGACATCTGTGTCC |
| --- | --- |
| hICAM1-R | GGGGTCTCTATGCCCAACAA |
| hVCAM1-F | GGGAAGATGGTCGTGATCCTT |
| hVCAM1-R | TCTGGGGTGGTCTCGATTTTA |
| hE-selectin-F | AGAGTGGAGCCTGGTCTTACA |
| hE-selectin-R | CCTTTGCTGACAATAAGCACTGG |
| hIL6-F | AAATTCGGTACATCCTCGACGG |
| hIL6-R | GGAAGGTTCAGGTTGTTTTCTGC |
| hIL1β-F | ATGATGGCTTATTACAGTGGCAA |
| hIL1β-R | GTCGGAGATTCGTAGCTGGA |
| hCCL2-F | CAGCCAGATGCAATCAATGCC |
| hCCL2-R | TGGAATCCTGAACCCACTTCT |
| hCCL5-F | CCAGCAGTCGTCTTTGTCAC |
| hCCL5-R | CTCTGGGTTGGCACACACTT |
| hCXCL5-F | AGCTGCGTTGCGTTTGTTTAC |
| hCXCL5-R | TGGCGAACACTTGCAGATTAC |
| hTNFα-F | CCTCTCTCTAATCAGCCCTCTG |
| hTNFα-R | GAGGACCTGGGAGTAGATGAG |
| hNEDD4L-F | GACATGGAGCATGGATGGGAA |
| hNEDD4L-R | GTTCGGCCTAAATTGTCCACT |
| mICAM1-F | GTGATGCTCAGGTATCCATCCA |
| mICAM1-R | CACAGTTCTCAAAGCACAGCG |
| mVCAM1-F | AGTTGGGGATTCGGTTGTTCT |
| mVCAM1-R | CCCCTCATTCCTTACCACCC |

**
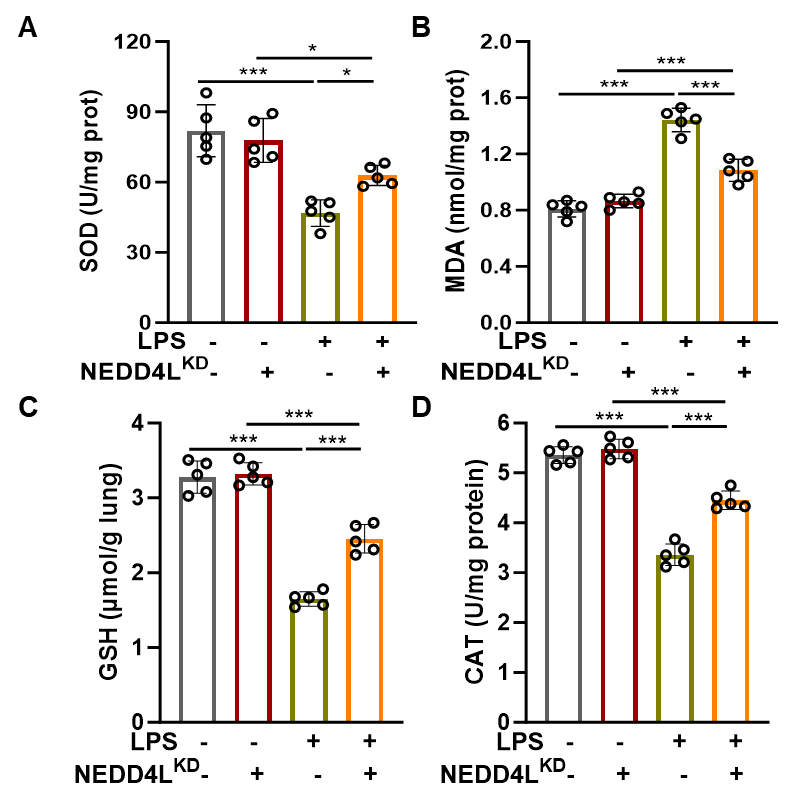
**

**Figure S1. NEDD4L knockdown alleviates LPS-induced oxidative stress responses in mouse lung tissues**

**A-D**. Quantification of oxidative stress-related parameters in lung tissues from WT and NEDD4L^KD^ mice with or without LPS stimulation: (A) superoxide dismutase (SOD) activity, (B) malondialdehyde (MDA) concentration, (C) glutathione (GSH) content, and (D) catalase (CAT) activity (n=5).


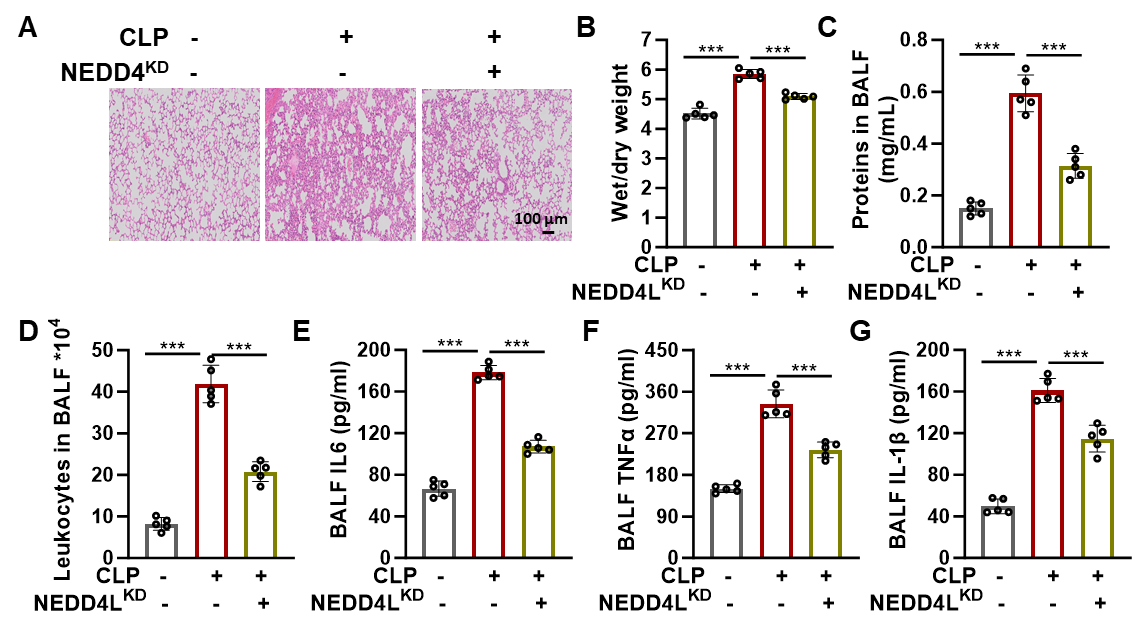


**Figure S2. Endothelial-specific knockdown of NEDD4L suppresses ALI induced by CLP surgery in mice**

**A.** H&E staining of lung sections from WT and NEDD4L^KD^ mice post-sham or CLP surgery (scale bar=100 μm).

**B.** Lung wet/dry weight ratio in each group, reflecting pulmonary edema severity (n=5).

**C-G.** Quantification of inflammatory parameters in BALF: total protein concentration (C), total leukocyte count (D), IL-6 (E), TNFα (F), and IL-1β (G) levels (n=5).


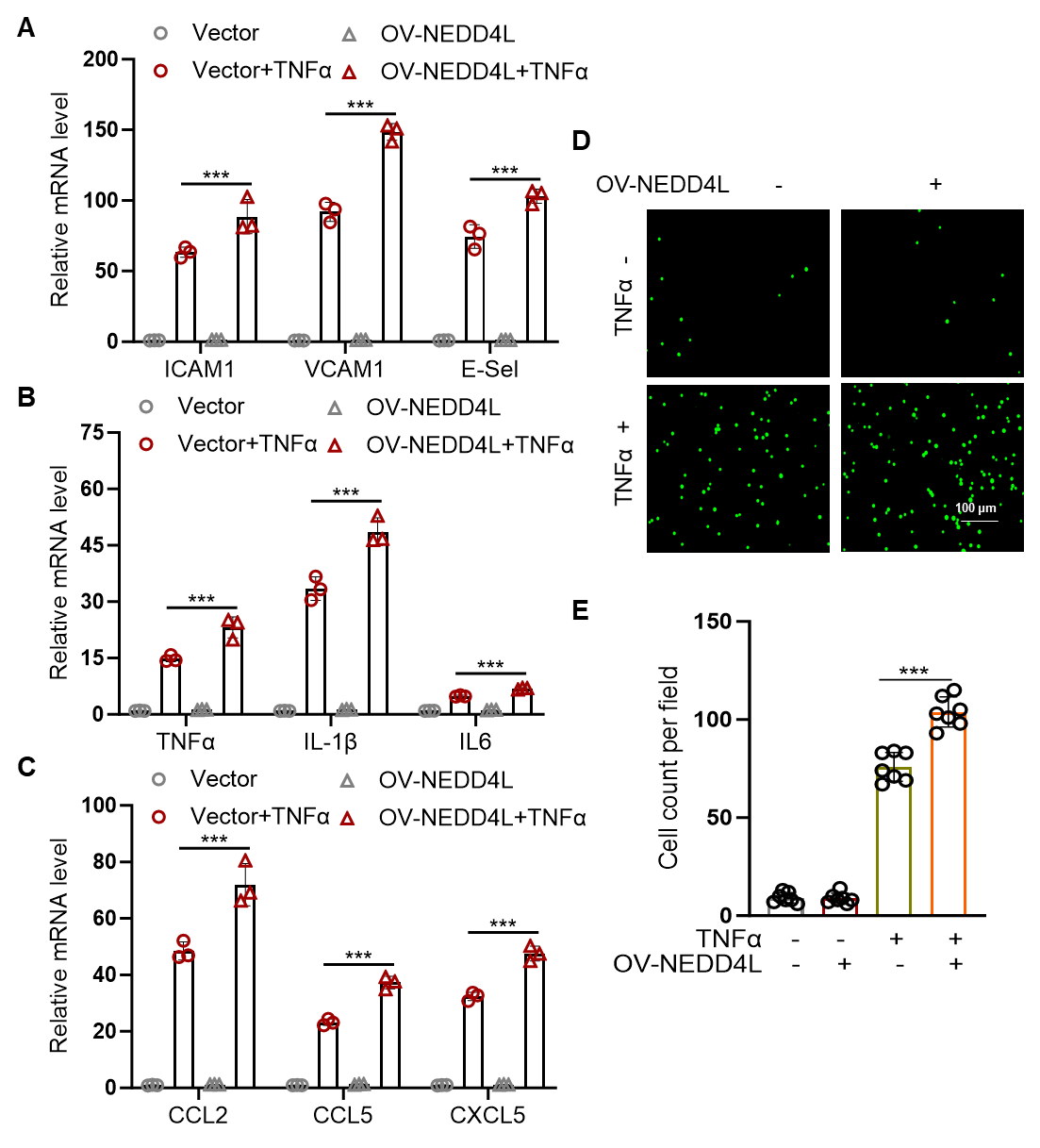


**Figure S3. Overexpression of NEDD4L potentiates TNFα-induced** **inflammatory and adhesion molecules expression in endothelial cells**

**A-C.** Relative mRNA levels of adhesion molecules (ICAM1, VCAM1, E-Selectin) (A), pro-inflammatory cytokines (TNFα, IL-1β, IL-6) (B) and chemokines (CCL2, CCL5, CXCL5) (C) in HUVECs transfected with empty vector or NEDD4L overexpression construct (OV-NEDD4L), with or without TNFα stimulation (n=3).

**D.** Representative fluorescent images of HL-60 cells (green) adhering to endothelial cells transfected with vector or OV-NEDD4L, with or without TNFα treatment (scale bar=100 μm).

**E.** Quantification of adherent HL-60 cells per field (n=7).


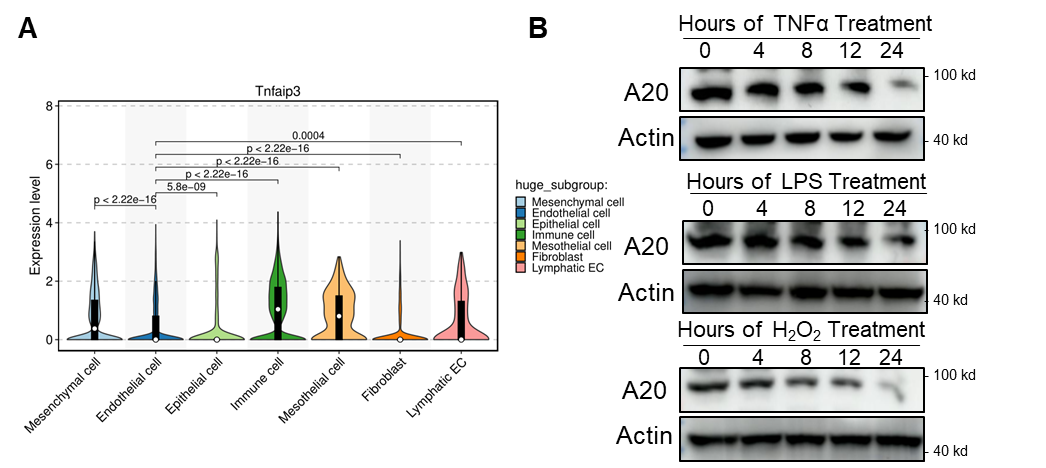


**Figure S4. A20 expression profiling under inflammatory and oxidative stress conditions**

**A.** Violin plots showing the distribution of A20 (Tnfaip3) expression across distinct cell clusters identified in the scRNA-seq dataset of GSE207651.

**B.** Western blot analysis of A20 protein expression in HUVECs treated with TNFα (10 ng/mL), LPS (1 μg/mL), or H₂O₂ (150 μM) over a 24 h time course. Actin serves as a loading control. Representative blots from three independent experiments are shown.


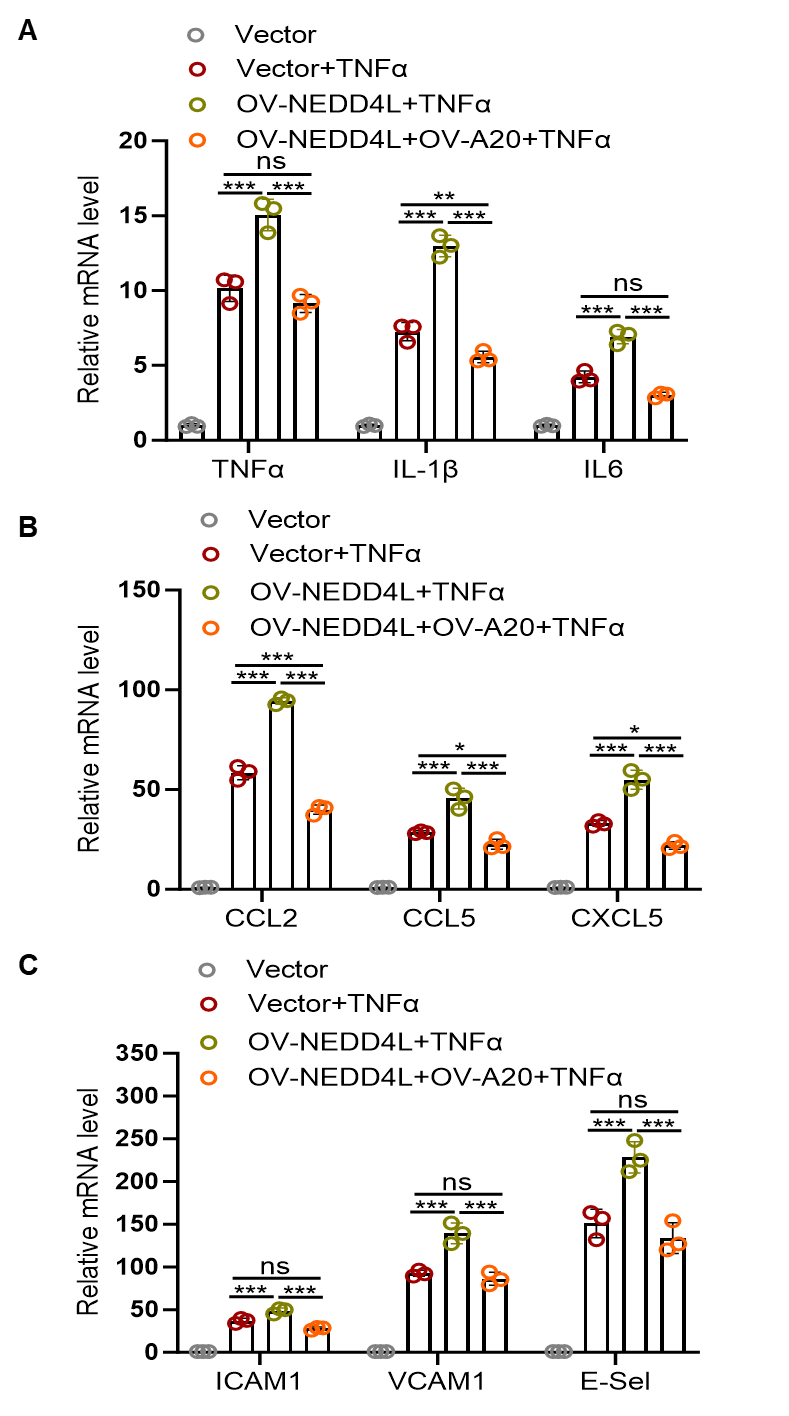


**Figure S5. A20 reverses NEDD4L-mediated potentiation of TNFα-induced inflammatory and adhesion molecules expression in endothelial cells**

A-C. Relative mRNA levels of pro-inflammatory cytokines (TNFα, IL-1β, IL-6) (A), chemokines (CCL2, CCL5, CXCL5) (B), and adhesion molecules (ICAM1, VCAM1, E-Selectin) (C) in HUVECs transfected with empty vector, OV-NEDD4L, or OV-NEDD4L plus OV-A20, with or without TNFα stimulation (n=3).


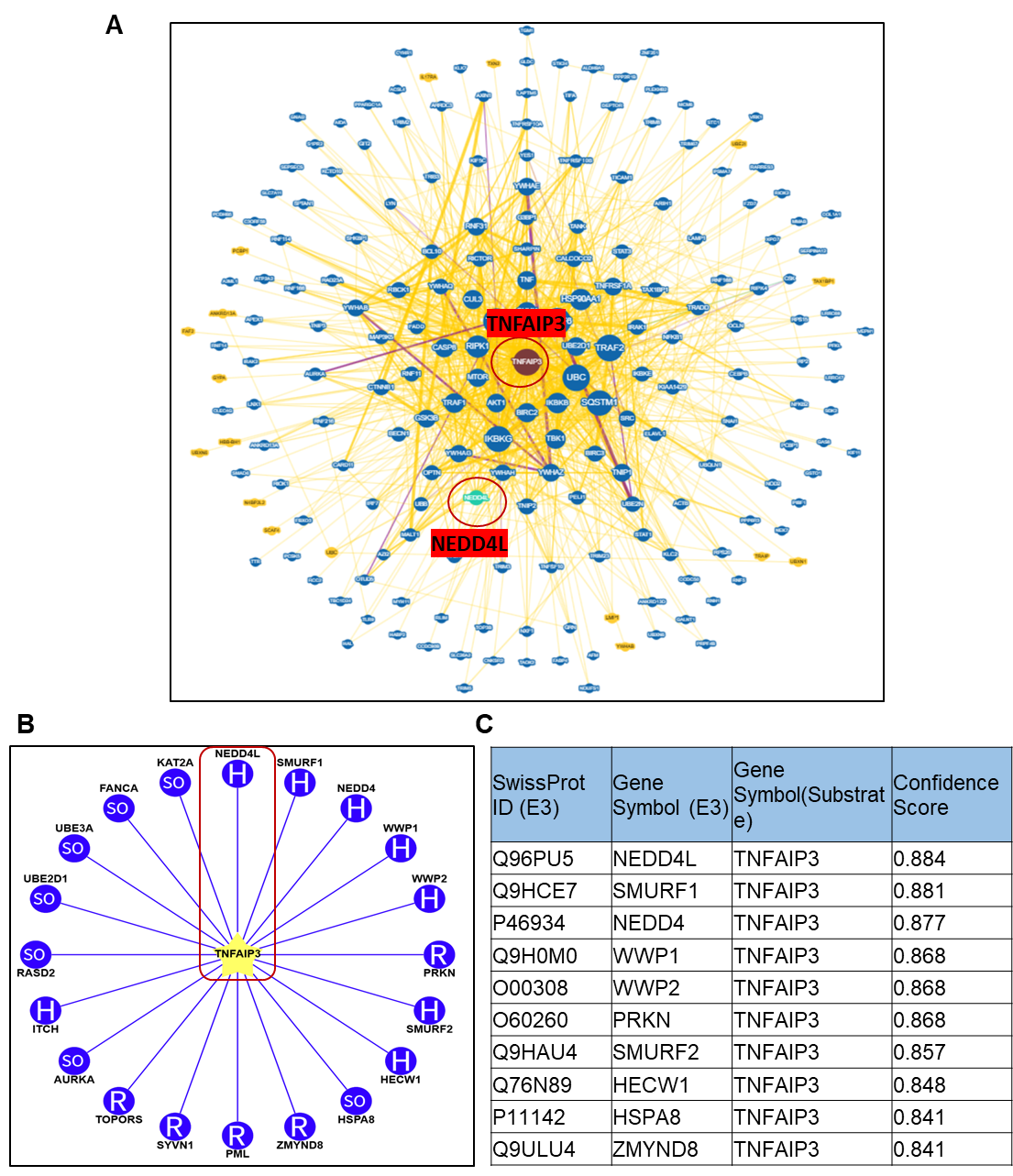


**Figure S6. NEDD4L is identified as a key E3 ligase interacting with A20 (TNFAIP3) via bioinformatic analysis**

**A.** Protein-protein interaction (PPI) network of A20 and its interacting partners, generated using the BioGRID database (<https://thebiogrid.org/>). A20 and NEDD4L are indicated by red circles.

**B.** E3 ligase interaction network centered on A20, constructed using the UbiBrowser database (http://ubibrowser.bio-it.cn/). NEDD4L is highlighted with a red box.

**C.** Top 10 E3 ligases with the highest confidence scores for interaction with A20, ranked by confidence score, extracted from the UbiBrowser network shown in (B).
