# Supplementary figures and images for: Endothelial NEDD4L exacerbates acute lung injury by targeting A20 for ubiquitination degradation
Source: Respir Res. 2026 Apr 6;27:222. doi: 10.1186/s12931-026-03655-w (PMC13248476; doi:10.1186/s12931-026-03655-w)

Fig 1I

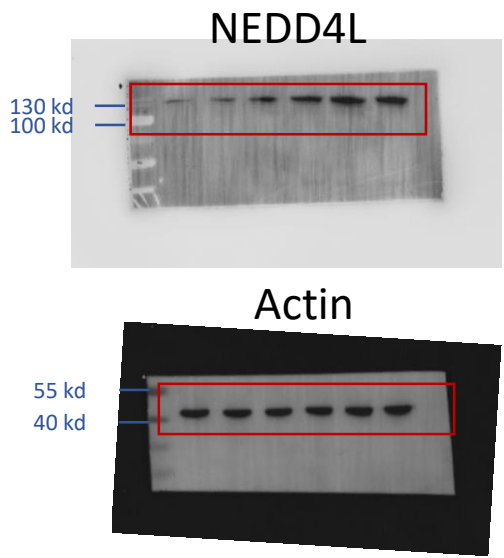

Fig 1J

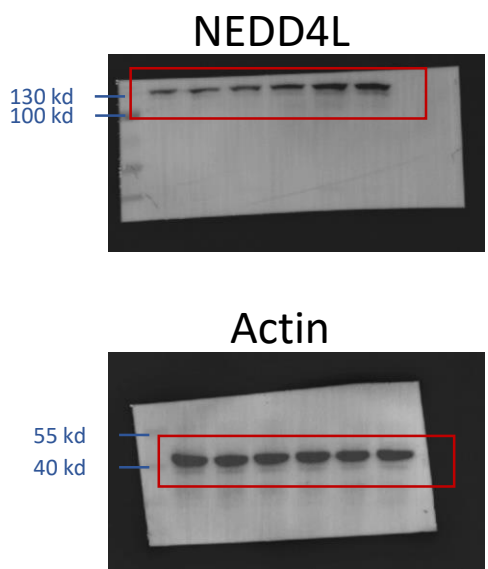

Fig 1K

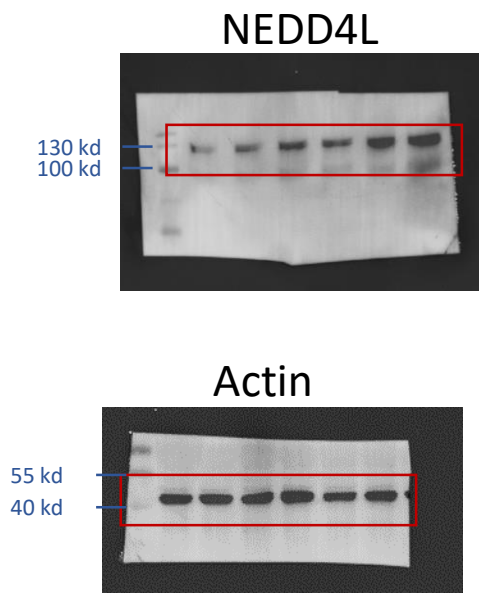

Fig 2A

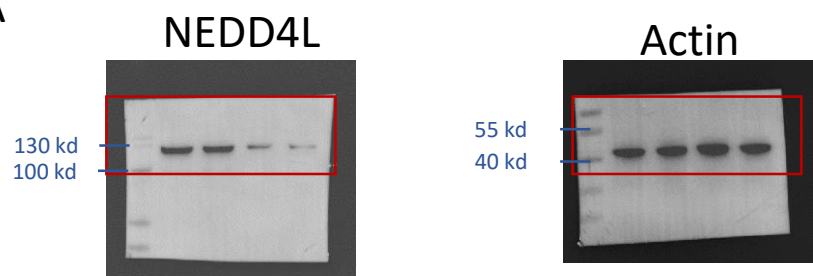

Fig 4A

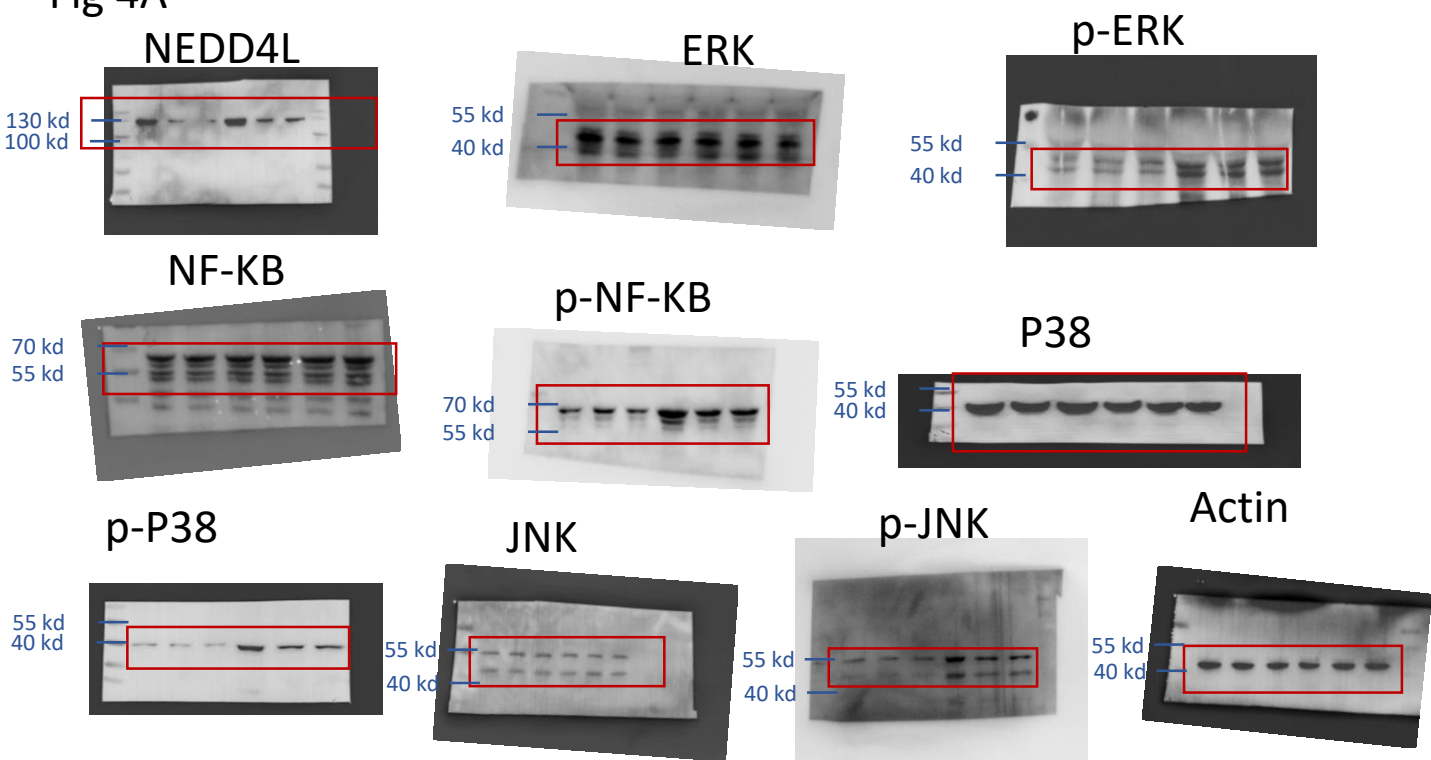

Fig 4C

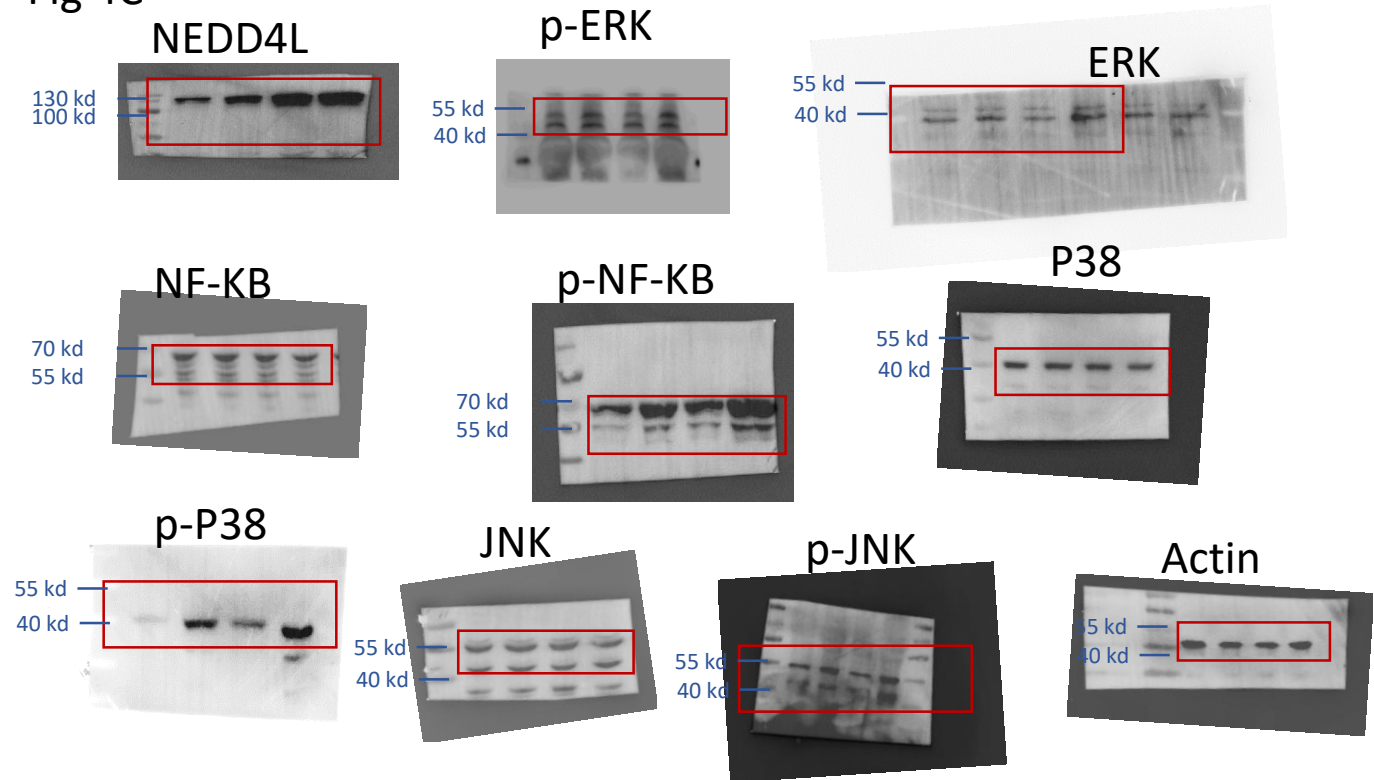

Fig 4E

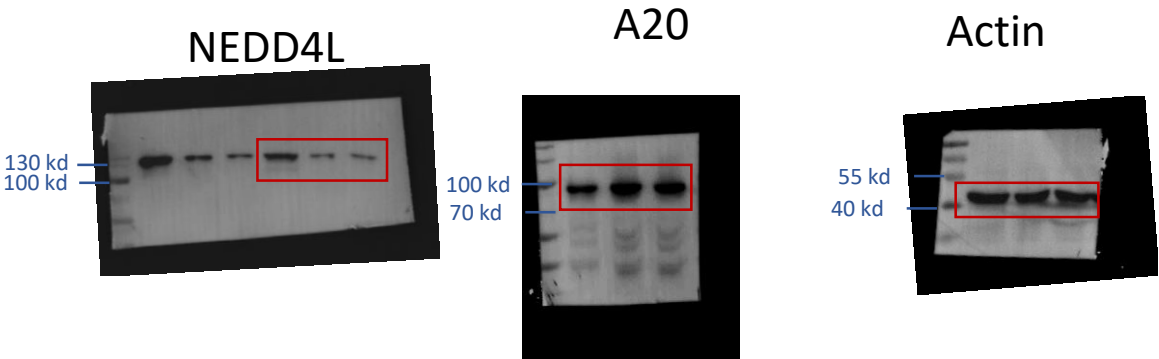

Fig 4G

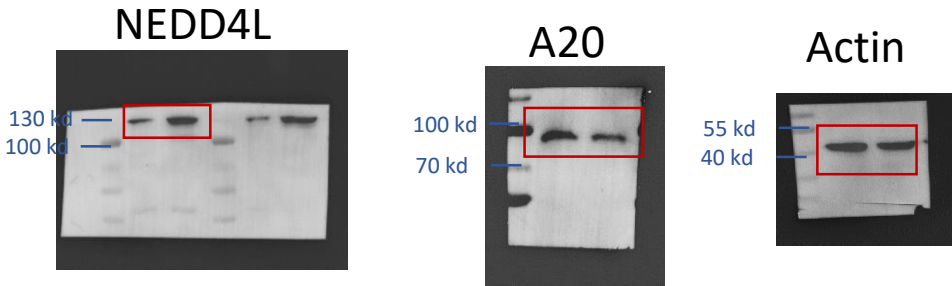

Fig 4H

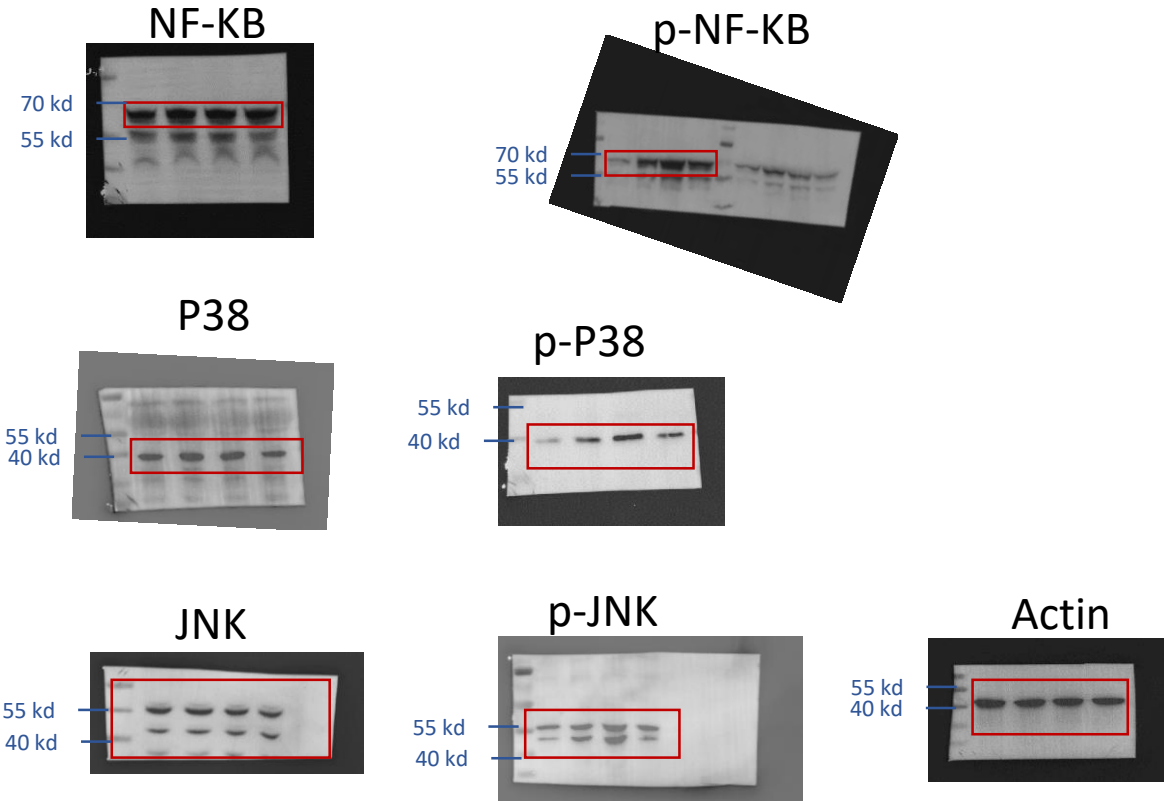

Fig 5B

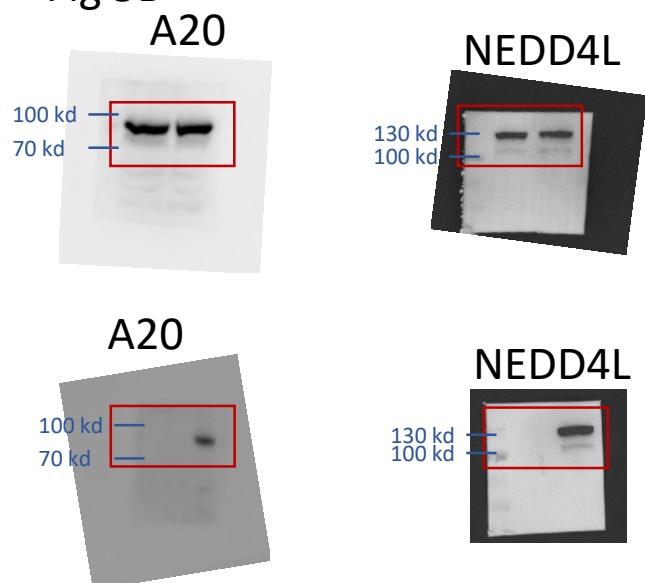

Fig 5C

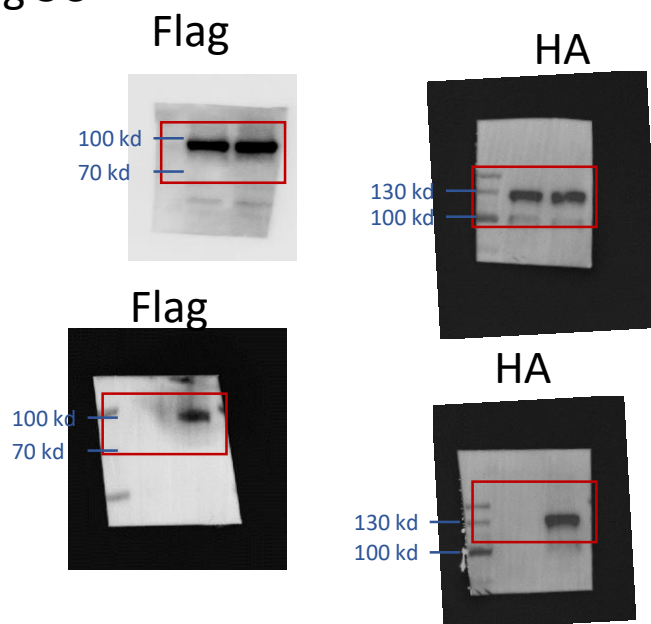

Fig 5D

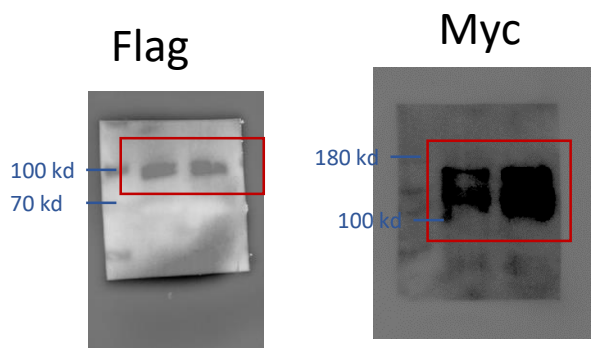

Fig 5E

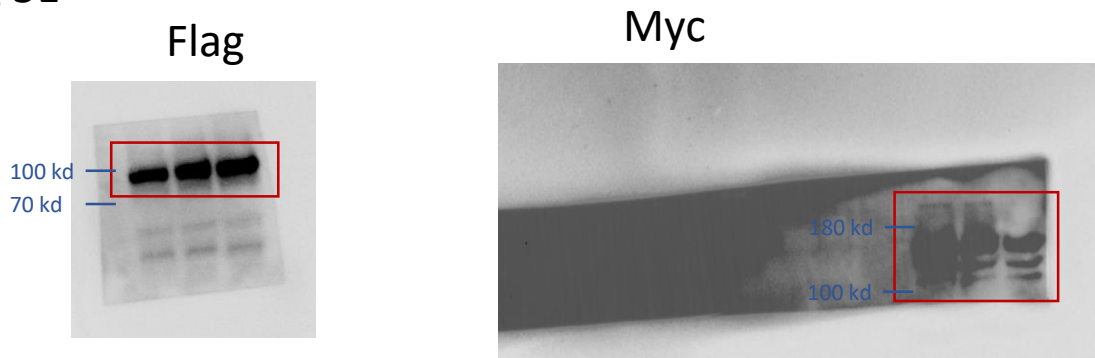

Fig 5F

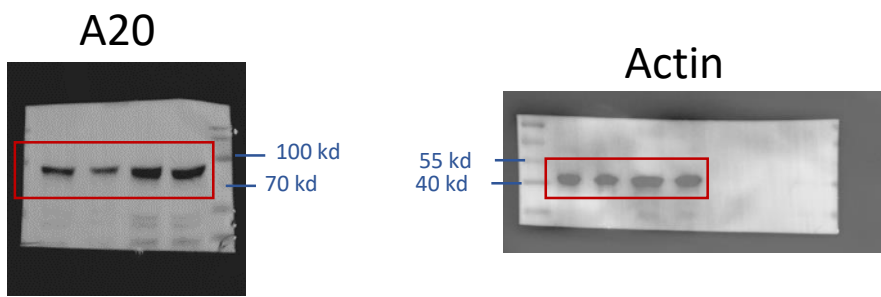

Fig S4A

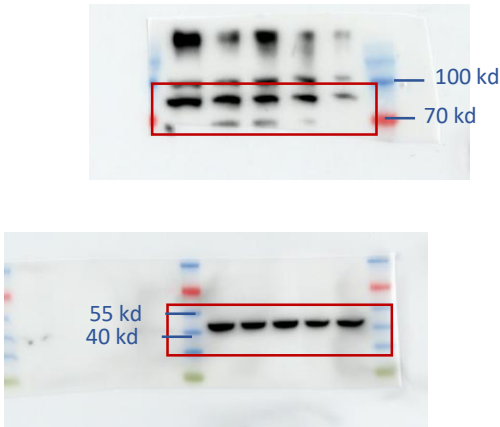

Fig S4B

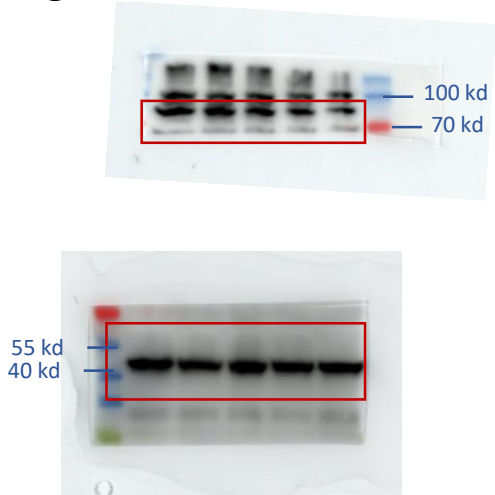

Fig S4C

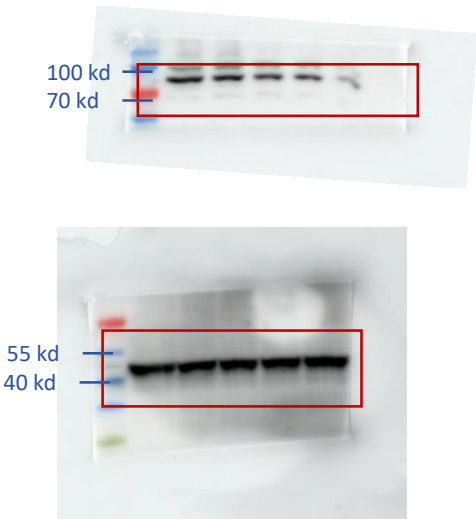

Supplement: Supplementary file 2 — Supplementary Material 2. [file 12931_2026_3655_MOESM2_ESM.pdf]
